# Kidney Disease Patient Representation in Trials of Combination Therapy With VEGF-Signaling Pathway Inhibitors and Immune Checkpoint Inhibitors: A Systematic Review

**DOI:** 10.1016/j.xkme.2023.100672

**Published:** 2023-05-16

**Authors:** Benjamin M.P. Elyan, Stephen Rankin, Rob Jones, Ninian N. Lang, Patrick B. Mark, Jennifer S. Lees

**Affiliations:** 1 School of Cardiovascular and Metabolic Health, College of Medical and Veterinary Life Sciences, University of Glasgow, Glasgow, UK; 2 NHS Greater Glasgow and Clyde, Glasgow, UK; 3 School of Cancer Sciences, College of Medical and Veterinary Life Sciences, University of Glasgow, Glasgow, UK

To the Editor:

Combination therapy with vascular endothelial growth factor signaling pathway inhibitors (VSPIs) and immune checkpoint inhibitors (ICIs) has led to major improvements in cancer survival. These survival improvements have not been observed to the same degree in people with chronic kidney disease (CKD) and cancer. CKD is highly prevalent in people with cancer^1^ and is associated with reduced survival in those diagnosed with some types of cancer.^2^ People with CKD have been poorly represented in cancer trials,^3^ and the evidence base for the use of ICI and VSPI in people with CKD is important because of their association with adverse kidney events.^4^ We assessed the extent to which people with markers of kidney disease are represented in clinical trials of combination therapy with ICI and VSPI.

We systematically searched MEDLINE, EMBASE, and Cochrane library databases (PROSPERO CRD42022337942) and followed Preferred Reporting Items for Systematic Reviews and Meta-analyses statement guidelines. The inclusion criteria used the PICO (population, intervention, comparison, and outcome) framework and included adult populations with any solid organ cancer receiving concurrent ICI and VSPI treatment in phase II-IV trials. Two reviewers independently assessed published articles and extracted data. The study did not require ethics approval and used a systematic narrative synthesis with quantitative analysis.

The primary outcomes of interest were: i) exclusion criteria related to kidney disease from trial protocols and ii) information about the representation of people with kidney disease in trials of combination therapy with ICI and VSPI.

Initial search identified 4,893 references, of which 32 trials spanning April 6, 2018 to December 4, 2022 and evaluating 11,066 participants met our pre-specified inclusion criteria. Most participants were assessed in Phase III trials (12 of 32 trials, 87.5% of participants); the remaining participants were assessed in phase II trials. There were 10 different combinations of ICI and VSPI. We could not obtain 1 trial’s full eligibility criteria (Zhang et al 2021, representing 0.3% of participants); this trial was excluded from the analysis (Table 1).

**Table 1:** Trials that met eligibility criteria, with trial characteristics and exclusion criterion used

Study	VSPI	ICI	Trial Population (n)	Tumor Site	Excluded With any Form of Kidney Disease	Exclusion Criterion Definition	Creatinine Clearance Cut-off (mL/min)	Were Patients with Proteinuria Excluded?	Was Baseline Kidney Function Available?	Were Patients on Immunosuppression Excluded?	Were Patients With Solid Organ Transplants Excluded
***Phase III randomized controlled trials***											
Motzer 2019	Axitinib	Avelumab	886	Renal cell	Yes	Creatinine clearance	50	Yes	No	Yes	No
Choueiri 2021	Cabozantinib	Nivolumab	651	Renal cell	Yes	Serum Creatinine in relation to upper limit of normal or Creatinine clearance	40	Yes	No	Yes	No
Colombo 2021	Bevacizumab	Pembrolizumab	617	Gynae	Yes	Serum Creatinine in relation to upper limit of normal or Creatinine clearance	60	Unspecified	No	Yes	No
Andre 2020	Bevacizumab	Pembrolizumab	307	Colorectal	Yes	Serum Creatinine in relation to upper limit of normal or Creatinine clearance	60	Unspecified	No	Yes	No
Makker 2022	Lenvatinib	Pembrolizumab	827	Gynae	Yes	Serum Creatinine in relation to upper limit of normal or Creatinine clearance	30	Yes	No	Yes	Yes
Moore 2021	Bevacizumab	Atezolizumab	1301	Gynae	Yes	Serum Creatinine in relation to upper limit of normal	NA	Yes	No	Yes	Yes
Motzer 2021	Lenvatinib	Pembrolizumab	1069	Renal cell	Yes	Creatinine clearance	30	Yes	No	Yes	Yes
Rini 2019 - keynote	Axitinib	Pembrolizumab	861	Renal cell	Yes	Serum Creatinine or Creatinine clearance	40	Yes	No	Yes	Yes
Finn 2020	Bevacizumab	Atezolizumab	501	Liver	Yes	Serum Creatinine or Creatinine clearance	50	Yes	No	Yes	Yes
Sugawara 2021	Bevacizumab	Nivolumab	550	Lung	Yes	Serum Creatinine or Creatinine clearance	50	Unspecified	No	Yes	Unspecified
Socinski 2018	Bevacizumab	Atezolizumab	1202	Lung	Yes	Serum Creatinine in relation to upper limit of normal	NA	Yes	No	Yes	Yes
Rini 2019 - Immotion	Bevacizumab	Atezolizumab	915	Renal cell	Yes	Measured or Creatinine clearance	30	Yes	No	Yes	Yes
***Phase II randomized controlled trials***											
Lheureux 2022	Cabozantinib	Nivolumab	82	Gynae	Yes	Serum Creatinine in relation to upper limit of normal or Creatinine clearance	50	Yes	No	Yes	Yes
Mettu 2022	Bevacizumab	Atezolizumab	133	Colorectal	Yes	Creatinine clearance	50	Yes	No	Yes	Yes
Nayak 2021	Bevacizumab	Pembrolizumab	80	GBM	Yes	Serum Creatinine in relation to upper limit of normal	NA	Yes	No	Yes	No
McDermott 2018	Bevacizumab	Atezolizumab	305	Renal cell	Yes	Serum Creatinine in relation to upper limit of normal or Creatinine clearance	40	Yes	No	Yes	Yes
Redman 2022	Bevacizumab	Avelumab	26	Colorectal	Yes	Creatinine clearance	30	Unspecified	No	Yes	Yes
***Phase II non-randomized multi-arm trials***											
Nayak 2022	Bevacizumab	Durvalumab	159	GBM	Yes	Serum Creatinine in relation to upper limit of normal or Creatinine clearance	50	Yes	No	Yes	Yes
Awada 2020	Axitinib	Avelumab	54	GBM	Yes	Creatinine clearance	30	Yes	No	Yes	No
***Phase II non-randomized single-arm trials***											
Cousin 2021	Regorafenib	Avelumab	46	Colorectal	Yes	Creatinine clearance	30	Yes	No	Yes	Yes
Cousin 2022	Regorafenib	Avelumab	34	Other	Yes	Creatinine clearance	30	Yes	No	Yes	Yes
Kawazoe 2020	Lenvatinib	Pembrolizumab	29	Gastric	Yes	Serum Creatinine in relation to upper limit of normal	NA	Yes	No	Yes	No
Lam 2021	Bevacizumab	Atezolizumab	40	Lung	Yes	Serum Creatinine in relation to upper limit of normal or Creatinine clearance	50	Yes	No	Yes	No
Lee C 2022	Cabozantinib	Nivolumab	47	Renal cell	Yes	Serum Creatinine in relation to upper limit of normal or Creatinine clearance	30	Yes	No	Yes	No
Lee J 2022	Bevacizumab	Atezolizumab	42	Lung	Yes	Creatinine clearance	30	Yes	No	Yes	No
Liu 2019	Bevacizumab	Nivolumab	38	Gynae	Yes	Serum Creatinine in relation to upper limit of normal or Creatinine clearance	60	Yes	No	Yes	No
Makker 2019	Lenvatinib	Pembrolizumab	54	Gynae	Yes	Serum Creatinine in relation to upper limit of normal or Creatinine clearance	40	Yes	No	Yes	Yes
McGregor 2019	Bevacizumab	Atezolizumab	60	Renal cell	Yes	Creatinine clearance	30	Yes	No	Yes	Yes
Seto 2022	Bevacizumab	Atezolizumab	39	Lung	Yes	Serum Creatinine	NA	Yes	No	Yes	Unspecified
Wilky 2019	Axitinib	Pembrolizumab	33	Other	Yes	Serum Creatinine in relation to upper limit of normal or Creatinine clearance	60	Unspecified	No	Yes	Unspecified
Zhang 2021	Lenvatinib	Pembrolizumab	38	Other	Unspecified	Unspecified	NA	Unspecified	No	Unspecified	Unspecified
Zsiros 2021	Bevacizumab	Pembrolizumab	40	Gynae	Yes	Creatinine clearance	60	Yes	No	Yes	Yes

Abbreviations: n, number; VSPI, VEGF-signaling pathway inhibitor; ICI, immune checkpoint inhibitor; GBM, Glioblastoma; Gyne, Gynecological cancers; NA, not applicable.

**Figure 1:**
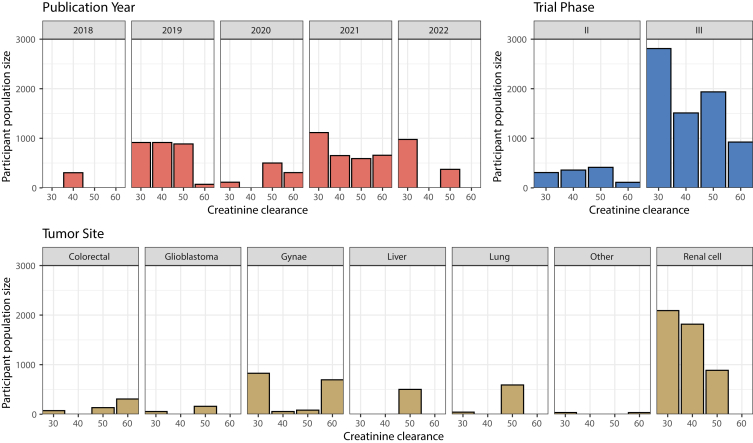
Bar graphs to demonstrate the creatinine clearance values used for exclusion from trials of combination therapy with VEGF-signaling pathway inhibitors and immune checkpoint inhibitors, presented according to participant population size.

All trials contained at least 1 exclusion criterion pertaining to kidney disease. Creatinine Clearance (CrCl) was the most common exclusion criterion, either alone or in combination with another criterion (26 of 31 trials, 75.7% of participants). No trials using the criterion CrCl included people with CrCl of <30 mL/min. The CrCl cut-off values were inconsistent by trial phase, tumor site, publication year, and agents used in combination (Figure 1, Fig S1-S3). Six trials (6 of 31, 17.2% of participants) accepted alternatives measures of glomerular filtration rate (GFR). Participants with evidence of proteinuria were excluded in 26 of 31 trials (85.9% of participants). Semi-quantitative detection on urinalysis (24 of 31 trials, 84.9% of participants) was the most used exclusion criterion, either alone (3 of 31 trials, 1.1% of participants) or in combination with quantitative methods. All trials excluded people on immunosuppressive therapy. No trial published participants’ baseline kidney function or proteinuria in the primary results article.

We found that all published trials of combination therapy with ICI and VSPI excluded people with evidence of kidney disease. No study included people with advanced CKD and few studies included people with proteinuria. The findings are concerning given that both drugs are associated with adverse kidney effects when used alone, and in combination.^4^ The under-representation of people with CKD in trials may undermine external validity of the trial and the generalizability of results.

The evidence for administration of VSPI or ICI in advanced CKD is mainly from published case series or retrospective analysis. The paucity of safety data may deny the access of people with CKD to effective anti-cancer therapy or unnecessarily expose them to excess risk of adverse effects. All identified trials excluded people treated with immunosuppressive medications; however, the use of these agents in kidney transplant recipients is increasing. A recent analysis demonstrated high rates of transplant rejection following ICI initiation.^5^

Concerns have been raised about heterogeneity regarding laboratory measurements used for cancer trial eligibility, including kidney function.^6^ The accuracy of creatinine-based GFR estimating equations is susceptible to several factors. Moreover, cancer patients may have reduced creatinine generation because of sarcopenia, leading to overestimation of GFR.^7^ Inaccuracies in GFR estimation could expose patients to potentially toxic doses or, conversely, to inadequate dosing of medications with reduced anti-cancer efficacy.

Renalism, the systematic undertreatment of people with CKD, is not unique to cancer therapies.^8^^,^^9^ Given that CKD is more common among older people, ethnic minorities, and those from socioeconomically deprived backgrounds,^10^ improving the evidence base of people with CKD is crucial in reducing health care inequalities.

Limitations to this review include its strategy to capture eligibility criteria for original trials, potentially missing post-licensing data or pre-trial safety data. We may not have captured efforts to report the representation of participants with kidney disease in secondary trial publications. We could not find 1 of 32 full trial protocols; however, this trial included only 0.3% of the total number of participants.

In conclusion, no trial included people with advanced CKD or kidney transplant recipients and few included people with proteinuria. Given CKD’s high prevalence in people with cancer and its association with worse cancer outcomes, targeted efforts should improve the representation of people with CKD in cancer trials to enhance external validity. Where exclusions are biologically justified, standardizing the approach using relevant markers of kidney function would improve the clinical application.
