# Supplementary material for: Kidney Disease Patient Representation in Trials of Combination Therapy With VEGF-Signaling Pathway Inhibitors and Immune Checkpoint Inhibitors: A Systematic Review
Source: Kidney Med. 2023 May 16;5(7):100672. doi: 10.1016/j.xkme.2023.100672 (PMC10363559; doi:10.1016/j.xkme.2023.100672)

Figure S1.
Prisma
Flowchart

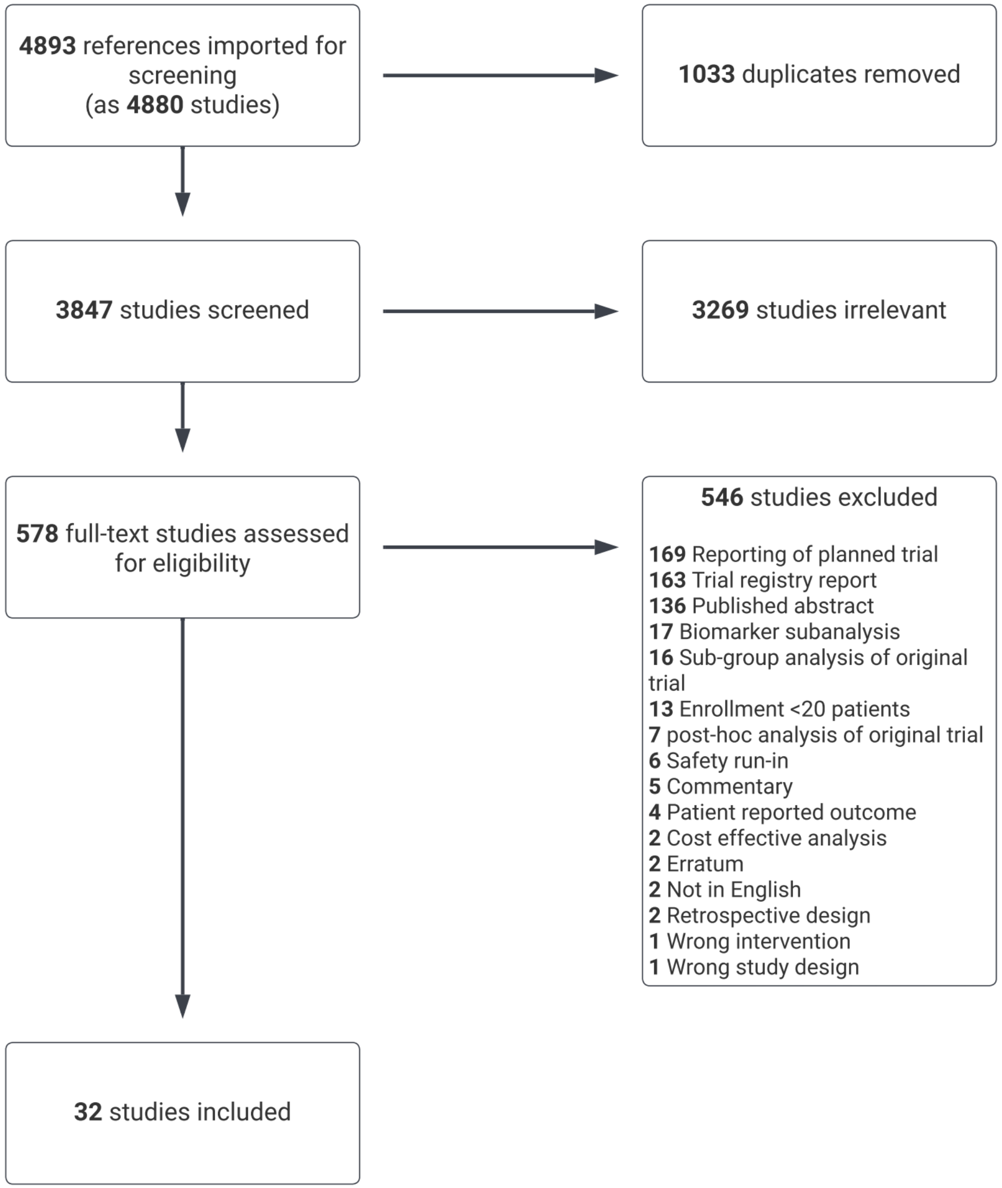

Figure S2. Bar plot of creatinine clearance cut-off values in trials by publication year, trial phase, and tumor site

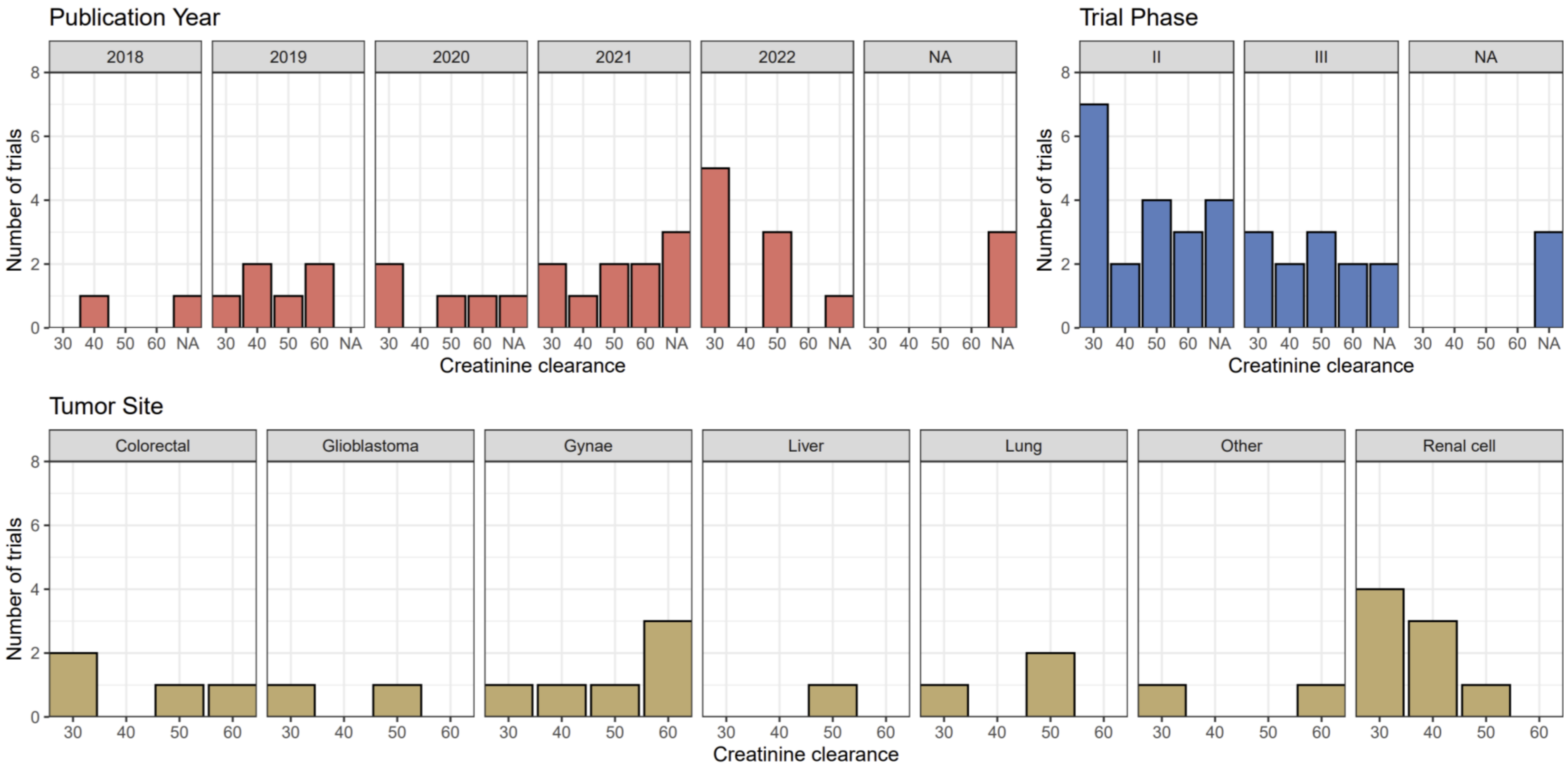

Figure S3. Heat map of creatinine clearance cut-off values and trial population size

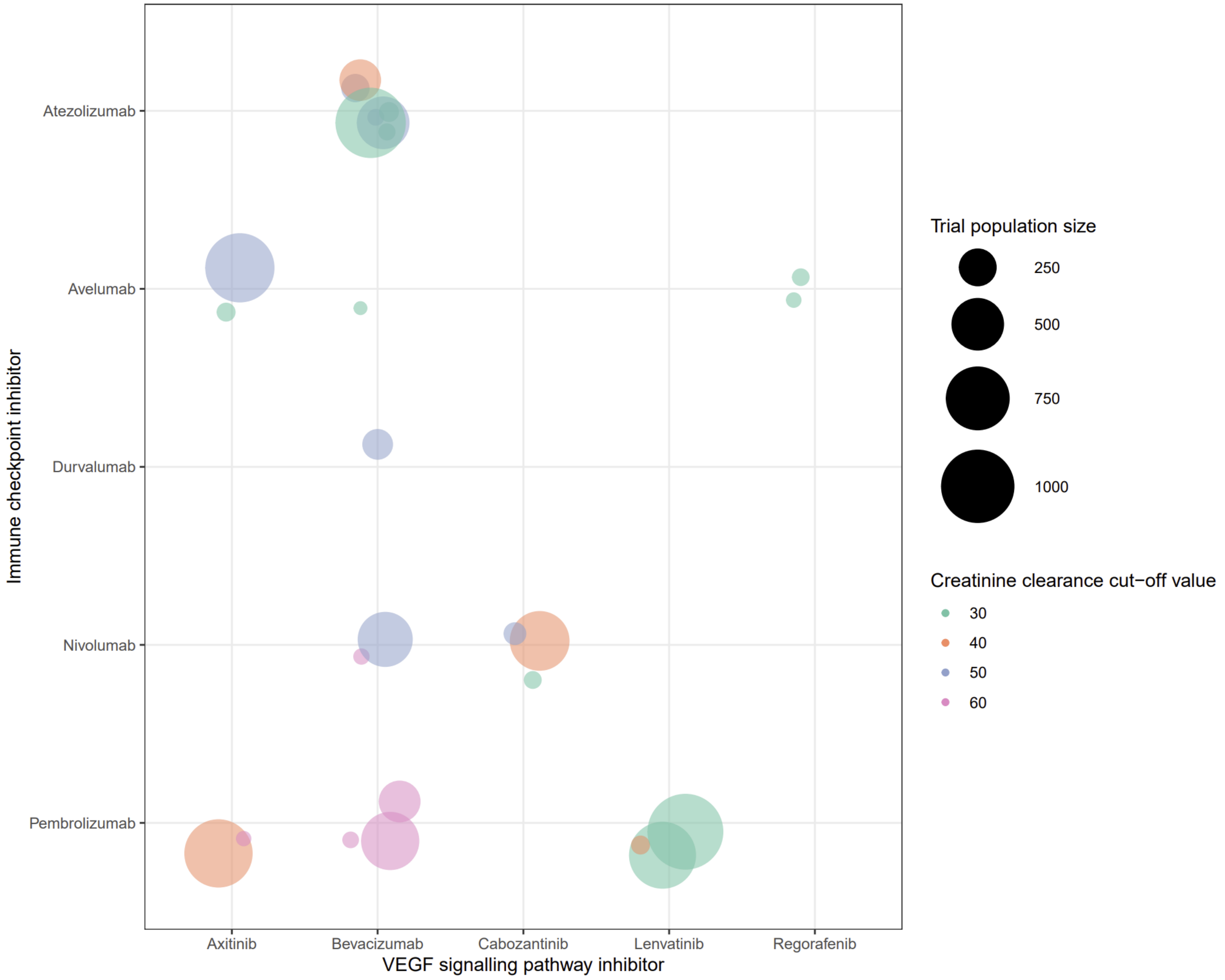

Supplement: Supplementary File (PDF) — Figure S1-S3. [file mmc1.pdf]
